# Modulation of the Neurovascular Unit by the Locus Coeruleus–Norepinephrine System: From Physiological Mechanisms to Therapeutic Applications

**DOI:** 10.1096/fj.202502069R

**Published:** 2025-10-13

**Authors:** Zixin Pan, Zhaoxing Jia, Tianxiang Jiang, Qian Cai, Zhong Di, Lin Gan, Congcong Ma, Xianming Lin

**Affiliations:** ^1^ The Third Clinical Medical College Zhejiang Chinese Medical University Hangzhou China

**Keywords:** blood–brain barrier, cerebral blood flow, locus coeruleus, neurovascular unit, norepinephrine

## Abstract

Neurovascular unit (NVU), a dynamic functional complex integrating neurons, glial cells, and cerebrovascular components, serves as the cornerstone for understanding brain pathophysiology. The locus coeruleus (LC)–norepinephrine (NE) system, through its extensive noradrenergic projections, critically modulates both cellular and systemic functions of the NVU. This review aims to systematically introduce the anatomical structure and functional characteristics of the LC–NE system and NVU, analyze the specific effects of the LC–NE system on NVU cellular components, explore the physiological mechanisms by which the LC–NE system regulates overall NVU function, and evaluate translational therapeutic applications targeting LC–NE pathways for NVU restoration. Deciphering the modulation of NVU by the LC–NE system not only provides novel insights into brain physiology but also opens new avenues for developing therapies targeting neurological disorders.

## Graphical abstract

The locus coeruleus (LC)–norepinephrine (NE) system significantly influences the structure and function of the neurovascular unit (NVU). The activation degree and firing patterns of LC–NE neurons, as well as the release amount and action sites of NE, profoundly impact the neurons, glial cells, and cerebrovascular components of the NVU. This comprehensive regulation includes modulating cerebral blood flow (CBF) and blood–brain barrier (BBB) permeability. Clinically, treatments that activate the LC–NE system have improved NVU dysfunction in various brain diseases.

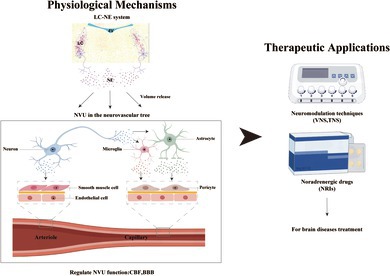

**Abbreviations**
6‐OHDA: 6‐hydroxydopamine
AAs: arachidonic acids
ARs: adrenergic receptors
BBB: blood–brain barrier
C1: catecholaminergic area
cAMP: cyclic adenosine monophosphate
CAMs: capillary‐associated microglia
CBF: cerebral blood flow
CCL2: the C‐C motif chemokine ligand 2
COX‐2: cyclooxygenase 2
CX3CL1: C‐X3‐C motif chemokine ligand 1
Cx43: connexin 43
DSP‐4: N‐(2‐chloroethyl)‐N‐ethyl‐2‐bromobenzylamine hydrochloride
EETs: epoxyeicosatrienoic acids
FDA: US Food and Drug Administration
FSS: fluid shear stress
GABA: γ‐aminobutyric acid
IL‐6: interleukin‐6
JAMs: junctional adhesion molecules
LC: locus coeruleus
mGluRs: metabotropic glutamate receptors
MMPs: matrix metalloproteinases
MRI: magnetic resonance imaging
NE: norepinephrine
NET: norepinephrine transporter
NMDA: N‐methyl‐D‐aspartic acid
NOS‐2: nitric oxide synthase
NPY: neuropeptide Y
NRIs: norepinephrine reuptake inhibitors
NVC: neurovascular coupling
NVU: neurovascular unit
PGE2: prostaglandin E2
PV: parvalbumin
rCBF: regional cerebral blood flow
RN: raphe nuclei
RVLM: rostral ventrolateral nucleus
SMCs: smooth muscle cells
SOM: somatostatin
TNF‐α: tumor necrosis factor‐alpha
TNS: trigeminal nerve stimulation
VIP: vasoactive long peptide
VNS: vagus nerve stimulation
ZO‐1: zona occludens 1

## 1 Introduction

The concept of the “neurovascular unit” (NVU) was formally proposed during the National Institute of Neurological Disorders and Stroke Review Group meeting on Stroke Progress in 2001 [1]. This complex and dynamic multi‐component system, consisting of neurons, glial cells, and cerebrovascular components, underscores the symbiotic relationship between neurons and cerebrovascular elements. Decades of research on the NVU have further elucidated the physiological regulatory mechanisms of the brain and the underlying pathophysiology of various brain diseases.

In the study of the structure and function of the NVU, it has been discovered that certain subcortical nuclei exert unique and profound influences on NVU's function through the secretion of neurotransmitters [2, 3, 4]. The locus coeruleus (LC)–norepinephrine (NE) system is one of the most extensively studied systems. Despite its small size, the LC is the primary source of NE in the brain. The noradrenergic neurons located in the LC release NE throughout the entire brain via an extensive fiber network, with all types of cells constituting the NVU serving as diffuse projection targets of the LC–NE system [5]. Additionally, pathological studies have revealed a correlation between abnormalities in the LC–NE system and NVU damages, observable in neurodegenerative diseases such as Alzheimer's disease and Parkinson's disease [6]. Degeneration of the LC–NE system often occurs in the early stages of these diseases, frequently preceding NVU lesions [7].

The modulation of the LC–NE system on the overall function of the NVU was extensively studied in the early days. Researchers implanted electrodes into the LC to simulate different firing patterns of LC noradrenergic neurons, observing changes in cerebral blood flow (CBF) and blood–brain barrier (BBB) permeability [8, 9]. By using drugs such as N‐(2‐chloroethyl)‐N‐ethyl‐2‐bromobenzylamine hydrochloride (DSP‐4) and 6‐hydroxydopamine (6‐OHDA) or physical methods like electricity or heat to destroy the LC, they further validated the modulation of the LC–NE system to the overall function of the NVU [8, 10, 11]. Although these early findings were controversial, advances in various blood flow imaging systems and the emergence of gadolinium‐enhanced MRI scanning have revealed detailed changes in CBF and blood–brain barrier permeability, illustrating the complexity of LC–NE system regulation of NVU function [12, 13]. In order to study how the LC–NE system regulates the structure of NVU, the use of norepinephrine‐related drugs to intervene in cell models in vitro is the most original research method [14, 15, 16, 17]. The emergence of calcium imaging, two‐photon imaging, and electrophysiological technology has promoted the in vivo study, and the short‐term physiological changes and interactions of cells have also been studied to a certain extent, which provides a mechanistic basis for elucidating the overall functional changes of NVU [3, 18].

In therapeutic practice, more and more attention has been paid to the intervention of the LC–NE system, especially electrical stimulation [19, 20]. These treatments have been proved to improve the dysfunction of the NVU and are widely used in the treatment of ischemic stroke, epilepsy, depression, and other brain diseases [21].

Building upon this foundation, the present review summarizes the basic anatomical structure and functional characteristics of the LC–NE system and NVU. Notably, this work discusses the effects of the LC–NE system on each component cell of the NVU for the first time, with a focus on the physiological and pathological changes of cells and their interactions. We further synthesize prior investigations elucidating LC–NE modulation of integrated NVU functionality while integrating cutting‐edge evidence to delineate the physiological mechanisms regulating CBF and BBB alterations. Finally, we evaluate translational therapeutic applications targeting LC–NE pathways, including neuromodulation and pharmacotherapeutic approaches for NVU functional restoration in brain diseases.

## 2 Anatomical Structure and Function of LC–NE System

**FIGURE 1:**
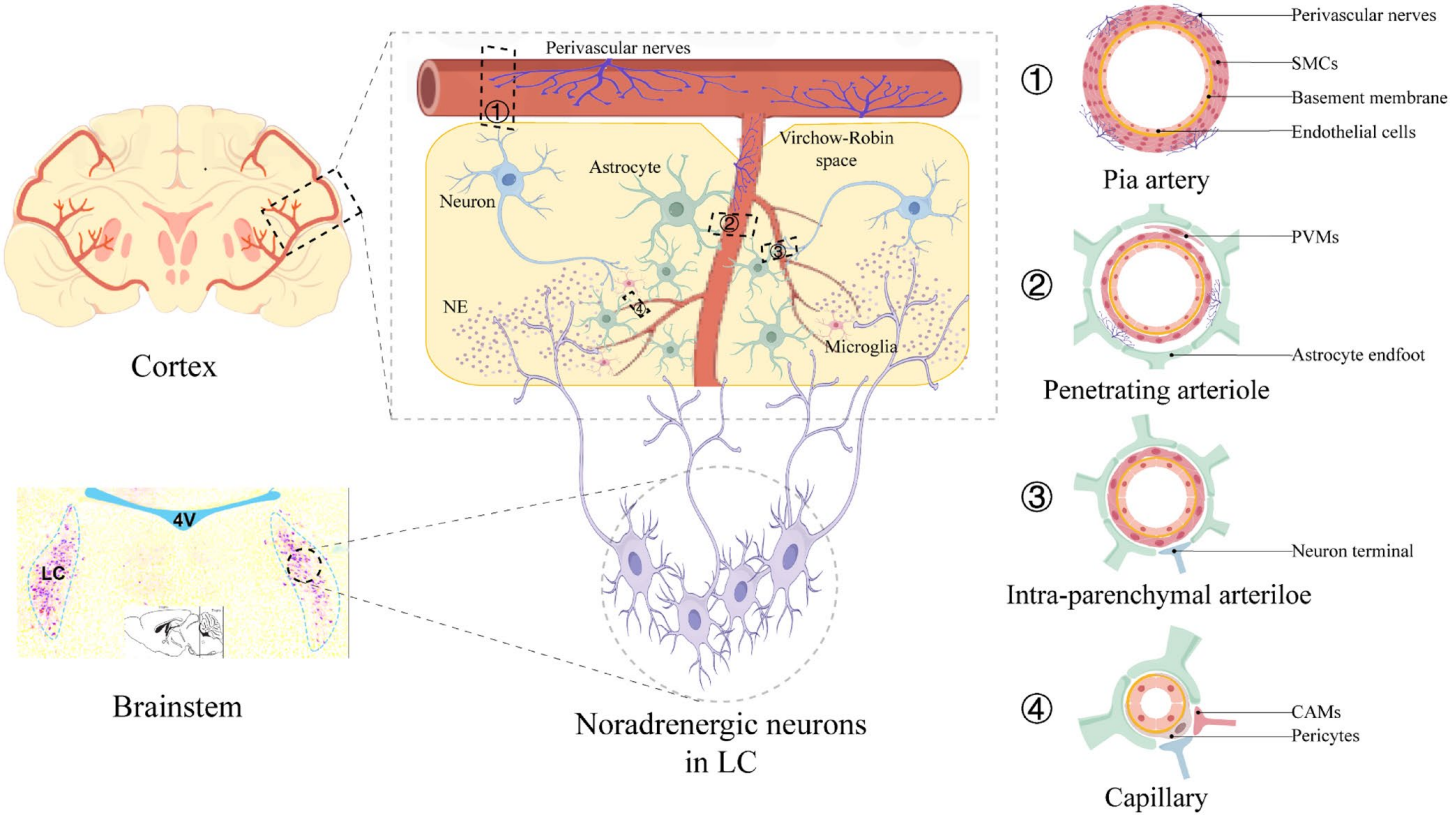
Anatomical structure of LC–NE system and NVU. LC is situated at the base of the fourth ventricle. The terminals of noradrenergic neurons originating from LC release NE through volume transmission. This released NE acts on neurons, neurogliocytes, and vascular components that collectively form NVU. CAMs, capillary‐associated microglia; LC, locus coeruleus; NE, norepinephrine; NVU, neurovascular unit; PVMs, perivascular macrophages; SMCs, smooth muscle cells.

The LC is a vital bilateral nucleus situated in the brainstem, specifically within the floor of the fourth ventricle, anterior, and posterior to the pons. It serves as the primary site for the synthesis of norepinephrine in the brain (Figure 1).

The LC is predominantly composed of noradrenergic neurons, with over half of the brain's noradrenergic neurons located in this region. These neurons synthesize and secrete norepinephrine, characterized by the expression of dopamine β‐hydroxylase, a crucial rate‐limiting enzyme in norepinephrine synthesis. Noradrenergic neurons within the LC are categorized into two main types: larger multipolar cells, primarily found dorsally, and smaller spindle cells, mostly located ventrally [22]. Recent research has revealed that LC neurons can also secrete other neurotransmitters such as neuropeptide Y (NPY) and dopamine, though their specific functions remain largely unknown [23, 24].

As the core of the brainstem reticular formation, the LC receives afferent inputs from various structures and projects diffusely throughout the brain via its noradrenergic neurons (Figure 1). Different regions, including the cerebellum, thalamus, and cortex, send direct inputs to the LC, with varying numbers of input neurons identified using transsynaptic rabies virus tracing methods [25]. Radioisotope injection studies have confirmed extensive projections from the LC to the anterior brain, cerebellum, brainstem, and spinal cord [26]. LC neurons projecting to forebrain regions like the hippocampus and septum are generally located dorsally. In contrast, those projecting to the cerebellum and spinal cord are positioned more ventrally. LC neurons targeting the hypothalamus are anterior, while those projecting to the thalamus are posterior. Neurons projecting to the cortex and amygdala are distributed throughout the LC [25, 27].

When LC noradrenergic neurons are excited, they release norepinephrine from their nerve terminals. This neurotransmitter acts on adrenergic receptors (ARs) of the postsynaptic cells, regulating their structure and function. The specific form of this regulation depends on the type and distribution of ARs expressed by the postsynaptic cells. Additionally, the firing rates of LC neurons play a crucial role in their modulatory effects. Optogenetic studies have revealed that tonic or phasic discharges of LC neurons promote wakefulness and alertness [28, 29]. In contrast, high‐frequency tonic discharges can induce anxiety‐like behaviors and aversive states in mice [30, 31]. These findings highlight the complex and nuanced ways in which LC activity can influence both physiological and behavioral outcomes.

## 3 Anatomical Structure and Function of NVU

NVU is a complex structure comprising neurons, glial cells (including astrocytes and microglia), and vascular components (including endothelial cells, pericytes, smooth muscle cells (SMCs), and the basement membrane) [32].

Along the neurovascular tree, the NVUs in different parts have different structures. The leptomeningeal arteries are located in the subarachnoid space and have thick layers of SMCs and endothelial cells forming their walls. They receive innervation from sensory ganglia, including sympathetic, parasympathetic, and trigeminal ganglia. These arteries descend further and become penetrating arterioles that traverse Virchow–Robin space, which is a perivascular space limited by glial cells in the brain parenchyma. Their SMC layer becomes thinner. Intraparenchymal arterioles are further subdivisions of penetrating arterioles, with walls made up of a single layer of SMCs and endothelial cells, wrapped by astrocyte endfeet. Some neuronal axons (such as local neurons and subcortical nucleus neurons) project around these blood vessels. The most downstream of the neurovascular tree is the capillary. It does not contain SMCs. Its walls are composed of a single layer of endothelial cells. However, its basement membrane contains pericytes. Capillaries are wrapped by astrocyte endfeet and some neuron axons [1, 32]. Capillary‐associated microglia (CAMs) also adhere to the capillaries, with some cell processes encircling them [33] (Figure 1).

The primary function of the NVU is to regulate CBF and BBB permeability [34]. Each cellular component plays a unique role. Activation of various types of neurons can cause and regulate changes in neurovascular coupling (NVC) [2, 35, 36]. Acting as intermediaries between neurons and vascular components, astrocytes receive and transmit signals from neurons. Through metabotropic glutamate receptors (mGluRs), they release arachidonic acids (AAs), prostaglandin E2 (PGE2), and epoxyeicosatrienoic acids (EETs) [37, 38, 39, 40]. These substances can be received by nearby vascular components, causing contraction or expansion of SMCs and pericytes, and altering the permeability of endothelial cells [41, 42, 43]. As regulators of brain activity, microglia primarily interact with other cells constituting the NVU through paracrine pathways. This interaction helps regulate NVU functions such as BBB permeability and CBF [44, 45].

## 4 Effects of LC–NE System on Components of NVU

**FIGURE 2:**
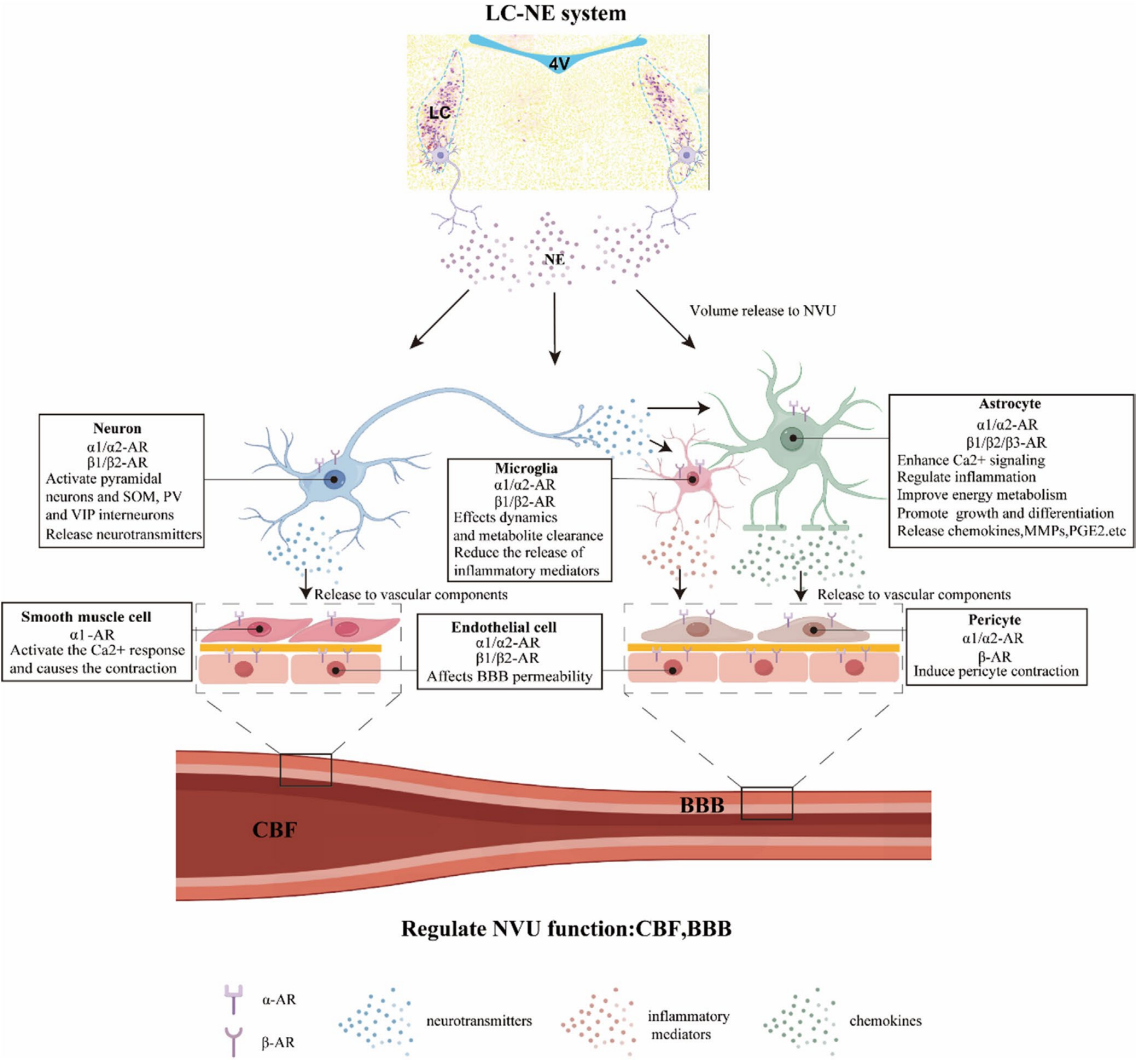
Effects of LC–NE system on components of NVU. LC–NE system releases NE through volume transmission, which acts on NVU. Neurons, neurogliocytes, and vascular components each express different adrenergic receptors. NE exerts varying effects by acting on these receptors. Neurons and neurogliocytes influence vascular components by secreting various mediators, while vascular components directly impact the NVU function. AR, adrenergic receptor; BBB, blood–brain barrier; CBF, cerebral blood flow; LC, locus coeruleus; MMPs, matrix metalloproteinases; NE, norepinephrine; NVU, neurovascular unit; PGE2, prostaglandin E2; PV, parvalbumin; SOM, somatostatin; VIP, vasoactive intestinal peptide.

LC is the primary source of NE in the neocortex. When LC noradrenergic neurons are activated, their nerve terminals release NE. Unlike the synaptic transmission of some neurotransmitters, NE is transmitted via volume transmission [46]. Consequently, NE can interact with a broad array of ARs expressed by various cells in the NVU, thereby modulating their structural and functional activities. ARs are classified into two main types: α‐adrenergic receptors (α‐ARs) and β‐adrenergic receptors (β‐ARs). The α‐ARs can be further subdivided into α1‐ARs and α2‐ARs. α1‐ARs are predominantly located at postsynaptic sites and are primarily coupled to the phospholipase C/inositol triphosphate/protein kinase C pathway, generally mediating excitatory effects. On the other hand, α2‐ARs are found at both presynaptic and postsynaptic locations. These receptors are negatively coupled to adenylyl cyclase, activating K^+^ currents and inhibiting Ca^2+^ channels, thus exerting an inhibitory function. The β‐ARs are specifically categorized into three subtypes: β1‐ARs, β2‐ARs, and β3‐ARs. The β1 and β2 isoforms are positively coupled to adenylyl cyclase, leading to an increase in cyclic adenosine monophosphate (cAMP), which directly or indirectly through protein kinase A‐triggered cascades affects synaptic excitability and plasticity. The specific role of β3‐ARs in the brain remains to be further elucidated [47]. The distribution patterns of noradrenergic receptors vary among different cells in the NVU, exhibiting differences in distribution types, density, and binding characteristics. As a result, the effects of the LC–NE system on these cells also differ accordingly [5] (Figure 2).

### 4.1 Neuron

Neurons express the α1‐AR, α2‐AR, β1‐AR, and β2‐AR isoforms [48]. Generally, the activation of the LC–NE system can enhance neuronal discharge, thereby promoting the function of NVU. Stimulating the LC–NE pathway recruits pyramidal cells and somatostatin (SOM), parvalbumin (PV) interneurons, as well as a small number of vasoactive long peptide (VIP) interneurons, leading to the activation and disinhibition of pyramidal cells [2]. Activation of these neuronal networks increases cortical activity and CBF through direct effects on blood vessels via cyclooxygenase 2 (COX‐2) and prostaglandin E2 (PGE2) or indirect effects on perivascular astrocytes [8]. The use of either N‐methyl‐D‐aspartic acid (NMDA) or γ‐aminobutyric acid (GABA) receptor antagonists blocks the cerebral vasodilatory response induced by LC stimulation, providing further evidence that glutamatergic neurons and GABA interneurons play a crucial role in regulating NVU function [8]. Additionally, LC is pivotal in functions such as attention, cognition, and sleep regulation by modulating neuronal activities [48, 49, 50]. The degeneration of the LC–NE system is associated with vascular and neuronal functional impairments in cognitive brain areas during the prodromal stage of neurodegenerative diseases, including Alzheimer's disease [49, 50].

### 4.2 Neurogliocyte

#### 4.2.1 Astrocyte

Astrocytes express all AR isoforms. Among these, the β‐ARs, particularly β2‐AR, are the most densely expressed subtypes in astrocytes [51, 52]. The terminals of LC noradrenergic neurons make contact with astrocytes in the cerebral cortex, where they influence the structure and function of astrocytes by releasing NE. This NE acts directly on the ARs expressed by astrocytes.

The LC–NE system influences astrocyte Ca^2+^ signaling and can trigger prolonged Ca^2+^ signals in astrocytes by acting on α1‐AR [53]. In diseases like Alzheimer's disease, where the LC–NE system is impaired, abnormal Ca^2+^ signaling in astrocytes is observed [54]. The increased cytoplasmic Ca^2+^ levels in astrocytes are noticeable in the cell body and endfeet [3]. In the endfoot, Ca^2+^ signaling induces the phospholipase A2‐arachidonic acid pathway, leading to the release of 20‐hydroxyeicosapentaenoic acid and subsequent cerebral vasoconstriction [55].

Moreover, the LC–NE system influences the synthesis and release of other mediators in astrocytes, especially inflammatory factors. By activating β‐ARs, NE can promote the synthesis of chemokines (CCL2) in astrocytes through increased cAMP levels, while activation of α2‐ARs inhibits this synthesis [56]. NE also induces the expression of interleukin‐6 (IL‐6) in astrocytes via the activation of α1‐ARs and β1‐ARs [57]. Additionally, NE can upregulate the expression of CX3CL1 and matrix metalloproteinases (MMPs) in astrocytes, thereby playing a significant role in inflammation regulation [14, 58].

The LC–NE system plays a crucial role in regulating inflammation. Under normal, non‐inflammatory conditions, it promotes the release of certain inflammatory factors and maintains their normal levels in the brain. In contrast, under inflammatory conditions, the LC–NE system exerts anti‐inflammatory effects [14]. For instance, in an LPS‐induced inflammatory environment, NE inhibits the release of inflammatory factors such as CX3CL1, CCL2, and TNF‐α, and reduces the expression of Ca^2+^‐independent nitric oxide synthase (NOS‐2) through β‐AR activation [14, 59, 60, 61]. Additionally, noradrenergic innervation significantly influences the degree of astrocyte activation following injury [62]. A1‐reactive astrocytes release inflammatory factors, which can cause damage to the blood–brain barrier [63]. By inhibiting the LC–NE system, the expression of A1‐reactive astrocytes increases [64]. Interestingly, under inflammatory conditions, NE amplifies the expression of COX‐2 and PGE2, which appears to support the restoration of cerebral perfusion and thus aids in tissue repair [14].

On the other hand, the LC–NE system affects the energy metabolism of astrocytes. NE can enhance glycolysis in astrocytes by activating α‐ARs and β‐ARs, and it triggers lactate release from astrocytes through β‐ARs activation [65, 66, 67]. Impairing the oxidative metabolism of astrocytes with the aconitase inhibitor fluorocitrate significantly reduces the CBF response induced by LC electrical stimulation [8].

Additionally, the LC–NE system influences astrocyte growth and differentiation by acting on β‐ARs [68, 69]. It also alters astrocyte plasticity through α1‐AR‐mediated regulation of connexin 43 (Cx43), which is essential for the formation and maintenance of the normal structure of NVU [70]. Notably, the LC–NE system's effects on astrocytes are not limited to NE as a mediator; it can also activate neurons to release related transmitters that act on astrocytes [71, 72].

#### 4.2.2 Microglia

Microglia express α1‐AR, α2‐AR, β1‐AR, and β2‐AR isoforms, with higher levels of β2‐AR compared to other brain cells [73, 74]. Activation of these receptors modulates microglial reactivity. Unlike astrocytes, NE reduces the synthesis and release of inflammatory mediators in microglia under both inflammatory and non‐inflammatory conditions. In vitro experiments show that NE upregulates cAMP via β2‐AR activation, inhibits the expression of pro‐inflammatory cytokines such as TNF‐α and IL‐6, and suppresses nitric oxide release from cultured microglia [17]. NE also inhibits the production of cytokines and chemokines induced by β‐amyloid in microglia and β‐amyloid co‐cultures. In vivo studies have confirmed that LC damage exacerbates the inflammatory state [75]. However, the LC–NE system has different effects on microglial activation across diseases. Knockdown and inhibition of β‐ARs reduce stress‐induced microglial activation in the brain, while LC–NE system activation inhibits microglial activation in neuropsychiatric systemic lupus erythematosus [76, 77]. The LC–NE system also affects microglial dynamics and related functions, generally inhibiting microglial migration and surveillance through β2‐AR activation [18, 78]. Conversely, in Alzheimer's disease models, NE increases microglial migration and phagocytosis of β‐amyloid [74]. In summary, microglia influence NVU function by regulating inflammatory states and metabolite clearance, but the effects of the LC–NE system on microglia and NVU remain controversial and warrant further investigation.

### 4.3 Vascular Components

#### 4.3.1 Endothelial Cell

Endothelial cells express α1‐AR, α2‐AR, β1‐AR, and β2‐AR isoforms [79]. The LC–NE system regulates endothelial cells and affects BBB permeability. In vivo experiments show that intrastriatal injection of NE in anesthetized rats increases the permeability and endocytosis of fluorescein sodium in endothelial cells, effects reversed by the α‐AR antagonist phenoxybenzamine [80]. In vitro, phenylephrine, an α‐AR agonist, enhances the permeability and endocytosis of sodium fluorescein in bovine brain capillary endothelial cells, while clenbuterol, a β‐AR agonist, has the opposite effect [15]. Thus, NE may increase BBB permeability by increasing brain capillary endothelial cell permeability and enhancing endocytosis via α‐AR activation. On the other hand, continuous NE depletion in the brain by 5 mg/kg noradrenergic neurotoxin reduces the expression of tight junction proteins occludin and zona occludens 1 (ZO‐1) between vascular endothelial cells, impairing BBB integrity [81]. Additionally, inflammatory mediators and other neurotransmitters released by neurons and glial cells can also affect capillary endothelial cell permeability, with cAMP potentially playing a role in modulating BBB function [82, 83, 84].

#### 4.3.2 Pericyte

Pericytes express α2‐AR and β‐AR isoforms [85]. Studies have shown that LC noradrenergic neuron terminals are closer to pericytes rather than arterioles. In acute brain slices, NE causes pericyte contraction without increasing cytosolic Ca^2+^ concentration, and the α1‐AR agonist phenylephrine does not induce contraction. Blocking α2‐AR greatly reduces NE‐induced pericyte contraction, while stimulating α2‐AR with xylazine or clonidine induces pericyte contraction. Therefore, NE‐induced pericyte contraction and capillary constriction are mediated via α2‐AR [86]. Another study found that excess NE in the cerebellum causes pericytes to contract capillaries, but glutamate reverses this effect [87]. This may explain the increased CBF following LC–NE system activation. Although NE can cause pericytes to contract capillaries, it also activates glutamatergic neurons to release glutamate, reversing this effect and ensuring capillary expansion for increased CBF.

#### 4.3.3 Smooth Muscle Cell (SMC)

SMCs mainly express α‐ARs. SMCs, the main components of arteries, are important regulators of vascular tension and blood flow distribution. In vitro experiments show that NE activates the Ca^2+^ response in SMCs via α1‐AR, causing contraction [16, 88]. Capillaries in metabolically active brain areas can transmit retrograde signals to precapillary arterioles, inducing the constriction and relaxation of SMCs in arterioles [89]. Not all brain tissue SMCs are innervated by noradrenergic neurons; for example, SMCs on leptomeningeal arteries and those at the choroid plexus are innervated by sympathetic nerve fibers from the superior cervical ganglion [79]. Additionally, the LC–NE system affects SMCs not only through NE but also by promoting glutamate release from glutamatergic neurons, with NMDA receptors expressed on SMCs. Glutamate acting on NMDA receptors can cause both relaxation and contraction of SMCs [90]. In conclusion, the relaxation and contraction of SMCs are influenced by multiple factors, and the regulatory effect of the LC–NE system on SMCs requires further study.

## 5 Effect of LC–NE System on Neurovascular Unit Function

The LC–NE system has complex and diverse effects on each component of NVU, and its regulation of NVU function is the comprehensive result of its effects on each component. Currently, there are two main methods to study the effect of the LC–NE system on NVU function:

1. Activation of the LC–NE System by LC Electrical Stimulation: Electrodes were implanted in LC to perform electrical stimulation with specific intensity and frequency. This method simulates different activation degrees and discharge patterns of LC noradrenergic neurons, thereby facilitating the study of the effects of LC–NE system activation on the nervous system and its physiological functions [8, 91, 92]
2. Disruption of the LC–NE System Using Drugs: N‐(2‐chloroethyl)‐N‐ethyl‐2‐bromobenzylamine hydrochloride (DSP‐4) is a selective neurotoxin that temporarily and specifically degrades LC noradrenergic fibers. The commonly used dose is 50 mg/kg given in two intraperitoneal injections one week apart. This method is currently the most common for interfering with the LC–NE system [8, 12, 93]. 6‐Hydroxydopamine (6‐OHDA) is a neurotoxin that destroys dopaminergic and noradrenergic neurons. Since it cannot cross the blood–brain barrier and does not specifically destroy LC noradrenergic neurons, it must be injected into the LC via stereotactic brain injection for precise destruction. The damage effect of 6‐OHDA is permanent and can be achieved in a relatively short time, making it suitable for experiments requiring long‐term disruption of the LC–NE system [94, 95]. Additionally, some studies have damaged LC by physical methods such as electricity and heat, but these methods are rarely used due to uncontrollable damage and danger [11].

### 5.1 Effect of LC–NE System on CBF

CBF is the amount of blood flowing through cerebral vessels per unit time. The earliest proposed and most fundamental function of NVU is to maintain a close coordination between brain functional activity and blood flow. This ensures that when neuronal activity increases, regional cerebral blood flow (rCBF) can correspondingly increase to meet the neuronal energy and metabolic demand, a process known as NVC [96]. Consequently, studying the effect of the LC–NE system on NVC and CBF has been a prevalent approach to investigate the regulation of the LC–NE system on the NVU. Over the past few decades, numerous studies have evaluated changes in CBF when the LC–NE system is activated and when it is damaged or inhibited. However, these results are highly variable and sometimes contradictory (Table 1). Broadly, the findings fall into three categories.

**TABLE 1:** Effect of LC–NE system on CBF.

Animal	Condition	LC–NE system intervention	Results	Refs.
C57BL/6 mice	Aldh1l1 Cre/ERT✖GCaMP6f	DSP‐4	No effect on functional hyperemia when all trials were combined In 40% trials, functional hyperemic responses↓ In the other 60% trials, vasodilation↑	[3]
C57BL/6 mice	Anesthetized	Atipamezole (α2‐AR antagonist)	The diameter of surface arteries↓ The duration of the hyperemic response↓	[12]
		DSP‐4	The duration of the hyperemic response↑	
Wistar rats	Epilepticus	LC stereotaxic lesion by 6‐OHDA	No significant differences for rCBF	[95]
SD rats C57BL/6 mice	Acute cortical slices of rats; NG2‐dsRed mice	Clonidine and xylazine (α2‐AR agonist)	In cortical slices, capillary constriction near pericytes↑	[86]
		Atipamezole (α2‐AR antagonist)	In cortical slices, capillary constriction↓ In vivo, dilation of capillaries near pericyte↑	
Cats	Spinalization	LC electrical stimulation (low frequency)	rCBF in the cortex, basal ganglia and white matter of the corpus callosum↓	[91]
		LC electrical stimulation (high frequency)	No changes for rCBF in any brain areas	
Cats	Anesthetized	LC electrical stimulation	rCBF↓	[92]
SD rats	Anesthetized	LC electrical stimulation	Cortical CBF↑	[8]
		LC electrical stimulation+DSP‐4	CBF responses↓	
		LC electrical stimulation+ Phentolamine (α‐AR antagonist)	CBF responses↓	
		LC electrical stimulation+ Propranolol (β‐AR antagonist)	CBF responses↓	
Cats	Anesthetized	LC electrical stimulation	The cerebral blood volume↓	[97]
Wistar rats	Hypercapnia	LC stereotaxic lesion by 6‐OHDA	No changes for the cerebrovascular response to hypercapnia	[10]
SD rats	Anesthetized	LC electrical stimulation	CBF in anterior brain regions↓	[98]
Cats	Anesthetized	LC stereotactic thermocoagulation lesion	No significant differences for CBF	[11]
		Propranolol (β‐AR antagonist)	CBF↓	
Wistar rats	Paralyzed	LC stereotaxic lesion by 6‐OHDA	No changes were detected	[99]
		NE ascending bundle electrothermic lesion	No changes were detected	

Abbreviations: 6‐OHDA, 6‐hydroxydopamine; AR, adrenergic receptor; CBF, cerebral blood flow; DSP‐4, N‐(2‐chloroethyl)‐N‐ethyl‐2‐bromobenzylamine hydrochloride; LC, locus coeruleus; rCBF, regional cerebral blood flow.

#### 5.1.1 Activation of the LC–NE System Can Reduce rCBF

Studies by Goadsby showed that low‐frequency (15 Hz) LC electrical stimulation can lead to a reduction in rCBF in the cortex, basal ganglia, and white matter of the corpus callosum in cats. Among these regions, the occipital cortex exhibited the most significant reduction, while the occipital colliculus did not show significant changes [91]. Additionally, Katayama found that LC pulsed stimulation (10–20 Hz, 30 s) similarly induced a persistent reduction in rCBF, suggesting that activation of the LC–NE system may have a constrictive effect on brain parenchyma vessels [92]. In a rat experiment by de la Torre, rCBF recorded after LC stimulation also showed a significant reduction in forebrain regions known to be innervated by ascending adrenergic pathways [98].

#### 5.1.2 Activation of the LC–NE System Can Increase rCBF

Toussay showed that unilateral LC stimulation evoked a greater increase in cortical CBF on the ipsilateral side than on the contralateral side in rats. These CBF responses were almost abolished by denervation with DSP‐4 and were significantly reduced by α‐AR and β‐AR antagonists [8]. It may explain this phenomenon by suggesting that norepinephrine released from LC noradrenergic axons generates vasoconstrictive tension in small arterial smooth muscle and capillary pericytes in some regions, thereby increasing cerebral blood perfusion and causing cerebral vasodilation in other regions [16, 86]. Besides ARs, Arribas demonstrated that nitric oxide plays a role in the vasodilatory effect of norepinephrine. They suggested that the impaired vasodilation in response to norepinephrine found in the basilar arteries of aged rats may be due to a reduction in nitric oxide production and/or release [100].

#### 5.1.3 The LC–NE System Had No Effect on CBF

Goadsby showed that activation of the LC–NE system had no effect on CBF. There was no change in CBF in any region of the cat brain in response to high‐frequency (50/s) LC electrical stimulation [91]. Reddy showed that disruption of the LC–NE system had no effect on CBF. CBF was not affected by bilateral stereotactic thermocoagulation of LC [11]. Additionally, many researchers have explored the effect of the LC–NE system on CBF in pathological states, such as epilepticus, hypercapnia, and paralysis, and concluded that the LC–NE system has no effect [10, 95, 99].

Based on the analysis of these research results, there may be the following reasons for these inconsistent conclusions:

1. Different activation and disruption methods of the LC–NE system. The parameters used for LC electrical stimulation vary, and low‐frequency and high‐frequency stimulation can lead to different conclusions. Early studies used stereotactic injection of 6‐OHDA or thermal damage, or intraperitoneal injection of various adrenergic receptor inhibitors to inhibit the LC–NE system. Recent studies mostly used DSP‐4.
2. Inconsistent CBF detection methods. The early studies mostly used the radioactive indicator dilution method and local tissue sampling to detect CBF, with relatively low detection accuracy, which could detect CBF in different brain regions. Recent studies have more precise detection methods, such as two‐photon imaging, which provides the possibility of real‐time observation of the various levels of blood vessels and surrounding cells in different brain regions.
3. Inconsistent CBF Detection Regions. Detecting different areas, such as the cortex, cerebellum, and the whole brain, can lead to different conclusions. This may be related to the heterogeneity of NVC in different brain regions that receive projections of the LC–NE system.
4. Diverse Animal Models. Early studies mostly used anesthetized cats or rats for research, and some studies used animal models in pathological conditions. Recent studies have begun to use awake mice and have used acute brain slices for research.

In addition, a broad model hypothesis may suggest that the LC–NE system optimizes CBF regulation under stress conditions, redistributing blood flow to active regions via NE‐mediated vasoconstriction [12]. This ensures that the most directly activated brain areas experience prioritized vasodilation and increased CBF, while other areas experience reduced CBF through cerebral vasoconstriction to maintain overall cerebral perfusion.

Recent studies support this hypothesis, demonstrating a “center‐surround effect” on CBF following sensory stimulation [101]. For instance, functional hyperemia “hot spots” appear in the core region of neuronal responses and dissipate with distance from this center. When sensory stimulation is performed in the hind limbs, NE‐enhanced animals exhibit a localized and short‐lived hyperemic response, whereas NE‐deficient animals show a more extensive and prolonged hyperemic response [12]. Furthermore, depletion of LC neurons with DSP‐4 alters sensory stimulation‐induced functional hyperemia in a region‐specific manner. The “hot spot” arterioles within the activation column may show attenuated dilation post‐DSP‐4 treatment, while surrounding arterioles might display enhanced dilation [3]. These findings collectively illustrate that the LC–NE system fine‐tunes CBF regulation under stress conditions, aligning blood flow swiftly and precisely with dynamic neural activity patterns and contributing to the spatiotemporal characteristics of hemodynamic responses.

### 5.2 Effect of LC–NE System on BBB

NVU forms the structural basis of BBB and plays a crucial role in its formation and maintenance. Among them, brain vascular endothelial cells with unique structural and functional characteristics constitute the most basic framework of BBB. These features include: expressing tight junctions and adhesion junctions, which strictly limit paracellular transport; lacking fenestrations, with low efficiency of caveolin‐mediated endocytosis; having low expression levels of leukocyte adhesion molecules, restricting the entry of peripheral immune cells into the brain parenchyma; expressing a variety of transporters, including efflux transporters and influx transporters [102]. Efflux transporters such as P‐glycoprotein and breast cancer resistance protein limit the transport of harmful substances or drugs into the brain [103]. Influx transporters ensure the transport of necessary substances into the brain, such as glucose transporter 1 that provides glucose and transferrin receptor that transports metal ions [104]. In addition, cells around the blood vessels such as pericytes and astrocytes also participate in the regulatory role of BBB. However, research into the regulatory role of the LC–NE system on BBB function is comparatively limited (Table 2). These results can be roughly divided into two categories:

**TABLE 2:** Effect of LC–NE system on BBB.

Animal	Condition	LC–NE system intervention	Results	Refs.
Wistar rats	Acute hypertension (angiotensin‐induced)	LC stereotaxic lesion by 6‐OHDA	No changes	[105]
	Acute hypertension (NE‐induced)		Albumin leakage↑	
	Seizures		Albumin leakage↑	
Wistar rats	Hyperkaliemia	LC stereotaxic lesion by 6‐OHDA	No Na^+^/K^+^ unbalance	[106]
Wistar rats	Epilepticus	LC stereotaxic lesion by 6‐OHDA	No abnormal BBB permeability to water	[95]
C57BL/6 mice	Parkinson's disease	Xamoterol (β1‐AR agonist)	BBB permeability↑	[107]
Wistar rats	Anesthetized	LC electrical stimulation	Mannitol permeability↑	[9]
Tg344–19 ad rats	Alzheimer's disease	LC stereotaxic lesion by DBH‐sap	BBB permeability↑	[49]
Wistar rats	Anesthetized	LC electrical stimulation (5, 15, 30 Hz)	In a frequency‐dependent manner, BBB permeability↑	[108]
		Phenoxybenzamine (α‐AR antagonist) +LC electrical stimulation (15 Hz)	Sodium fluorescein permeability increase↓	
		Pindolol (β‐AR antagonist) +LC electrical stimulation (5 Hz)	Sodium fluorescein permeability increase↑	
SD rats	Acute hypertension (angiotensin‐induced)	LC stereotaxic lesion by 6‐OHDA	125 I‐labeled albumin leakage↑	[109]
	Acute hypertension (adrenaline‐induced)		No changes	
NMRI mice	Anesthetized	DSP‐4	No changes for HRP leakage Water permeability↑	[110]
Long Evans rats	Anesthetized	Isoproterenol	14C‐alfa‐aminoisobutyric acid permeability↑	[111]
		Timolol (β‐AR antagonist)	14C‐alfa‐aminoisobutyric acid permeability↓	
SD rats	—	DSP‐4	TJ proteins (occludin and ZO‐1)↓	[81]
Bovine	Brain endothelial cells monolayer	NE	NaF leakage and pinocytosis↑	
		Phenylephrine (α‐AR agonist)	NaF leakage and pinocytosis↑	[15]
		Clenbuterol (β‐AR agonist)	NaF leakage and pinocytosis↓	

Abbreviations: 6‐OHDA, 6‐hydroxydopamine; AD, Alzheimer's disease; AR, adrenergic receptor; BBB, blood–brain barrier; DBH‐sap, dopamine β‐hydroxylase‐saporin; DSP‐4, N‐(2‐chloroethyl)‐N‐ethyl‐2‐bromobenzylamine hydrochloride; HRP, horseradish peroxidase; LC, locus coeruleus; NAF, sodium fluoride; NE, norepinephrine; TJ, tight junction; ZO‐1, zonula occludens‐1.

#### 5.2.1 The LC—NE System Help Maintain BBB Integrity

Studies indicate that the LC–NE system helps preserve BBB integrity under pathological conditions characterized by increased circulating catecholamines, such as hypertension, epilepsy, and neurodegenerative disease [49, 95, 105, 107]. For instance, disruptions to the LC–NE system via drugs can exacerbate BBB leakage, potentially due to compromised tight junctions and reduced Na^+^/K^+^ ATPase activity [81, 112]. The LC–NE system damage also leads to brain edema, especially in the cerebellum [110].

#### 5.2.2 Activation of the LC–NE System Can Increase the BBB Permeability

LC electrical stimulation at frequencies of 5, 15, and 30 Hz has been shown to enhance BBB permeability in a frequency‐dependent manner [108]. However, the increase in BBB permeability diminishes following short‐term repeated stimulation of the LC [9]. Intraventricular injection of NE can also mimic LC–NE system activation and increase BBB permeability [80]. Additionally, NE treatment in an in vitro BBB model has been demonstrated to increase BBB permeability [15].

Despite these observations, the specific site of action for the LC–NE system in enhancing BBB permeability remains controversial. Some studies have indicated that the LC–NE system acts on α‐ARs to increase BBB permeability, whereas stimulation of β‐ARs appears to have the opposite effect [15, 108]. Conversely, other research has shown that β‐AR agonists can increase BBB permeability [107, 111]. Mechanistically, the increase in BBB permeability induced by NE seems to be partly due to heightened pinocytosis activity in endothelial cells [15, 80].

It is evident that the LC–NE system exerts a dual regulatory function on BBB permeability, and its mechanisms are multifaceted. One promising approach may be to investigate the LC–NE system's role in modulating BBB permeability through its influence on CBF, which in turn affects fluid shear stress (FSS)—a known regulator of BBB structure and function. Changes in CBF alter FSS, which can either protect or damage the BBB depending on whether FSS levels are optimal, too high, or too low. FSS regulates the expression and distribution of junction proteins, influencing BBB structure [113]. Therefore, future research should explore whether the bidirectional regulation of BBB permeability by the LC–NE system is intertwined with its optimization of NVU‐mediated CBF regulation. Investigating how LC–NE system modulation impacts CBF and subsequently FSS could provide deeper insights into maintaining BBB integrity and controlling its permeability. Understanding these mechanisms may open new therapeutic avenues for diseases where BBB dysfunction plays a critical role.

## 6 Therapeutic Application of Regulating NVU by LC–NE System

Impairments in the LC–NE system are closely associated with a variety of brain disorders, including neurodegenerative diseases (such as AD), cerebrovascular diseases (such as cerebral microangiopathy), and psychiatric diseases (such as depression) [114, 115, 116]. These conditions not only significantly diminish patients' quality of life but also impose a substantial medical burden on society. Research indicates that pathological markers for neurodegenerative diseases, such as Tau and α‐synuclein, initially appear in LC [117, 118]. The degree of LC disruption was also associated with the severity of cortical pathology, cognitive and behavioral impairment, and the risk of clinical progression [119, 120]. Additionally, damage to the structure or function of NVU is a common pathological feature in these disorders [121, 122]. For instance, a decrease in CBF and disruption of BBB are observed in AD progression [123, 124]. Given this context, the clinical application of the LC–NE system to modulate the NVU has become a key focus of current medical research (Tables 3 and 4). Several products that influence LC–NE system activity have already been introduced into clinical use, with preliminary results showing improvements in NVU structural dysfunction across various neurological diseases [122, 123, 124]. Unfortunately, there is currently no direct evidence to prove that these therapies exert the improvement function on NVU through the LC–NE system, and the relevant mechanism research still needs to be further carried out.

**TABLE 3:** Clinical application of regulating NVU by LC–NE system.

Study design	Subjects	LC–NE system intervention	Intervention parameters	Evaluation method	Results	Refs.
Randomized controlled trial	11 Partial epilepsy patients	High stimulation VNS Low stimulation VNS	500 μs; 30 Hz; 30 s ON and 5 min OFF 130 μs; 1 Hz; 30 s ON and 3 h OFF	PET with intravenous [15O] H_2_O	rCBF ↑ in bilateral thalami, hypothalami, inferior cerebellar hemispheres, and right postcentral gyrus seizure frequency↓	[125, 126]
Non‐randomized control trial	10 Depressive patients	Acute VNS	0.25 mA; 0.5 mA; 0.75 mA	Functional transcranial doppler	No significant differences for CBF	[127]
Randomized controlled trial	20 Healthy volunteers	Transcutaneous auricular VNS	20 μs; 20 kHz; 1.5–3.8 mA	Arterial spin labeling MRI scans	Sustained CBF ↓ in the bilateral posterior cerebellum	[128]
Non‐randomized control trial	21 Refractory epilepsy patients	Pairing VNS	1.5–3.0 mA	Near‐infrared spectroscopy with sensors	CBF ↑ when a verbal fluency task was paired with VNS in a stimulation intensity‐dependent manner	[129]
Randomized crossover study	16 Healthy volunteers	Electroacupuncture TNS	0.25 ms; 100 Hz; 2.5–4.5 mV; 1 min ON and 1 min OFF; 11 min	Two‐channel near‐infrared spectroscope	rCBF ↑ in bilateral prefrontal cortex	[130]
Non‐randomized control trial	10 Drug‐resistant epilepsy patients	TNS	0.25 ms; 120 Hz; 30 s ON and 30 s OFF; 20 min	Single photon emission computed tomography	CBF↑ in cortex, namely in the temporal and limbic lobes	[131]
Randomized controlled trial	15 Healthy volunteers	Atomoxetine	60 mg p.o.	Arterial spin‐labeling MRI scans	rCBF ↓ in midbrain/substantia nigra and thalamus rCBF ↑ in cerebellar cortex	[132]
Case report	Attention deficit hyperactivity patient	Atomoxetine	—	Single photon‐emission computed tomography	rCBF ↑ in prefrontal cortex	[133, 134, 135]

Abbreviations: CBF, cerebral blood flow; MRI, magnetic resonance imaging; p.o., oral administration; PET, positron emission tomography; rCBF, regional cerebral blood flow; TNS, trigeminal nerve stimulation; VNS, vagus nerve stimulation.

**TABLE 4:** Preclinical application of regulating NVU by LC–NE system.

Animal	Condition	LC–NE system intervention	Results	Refs.
BALB/c mice	Traumatic brain injury	VNS	BBB permeability↓ AQP‐4↑	[136]
SD rats	Kindled rats with cortical dysplasia	VNS	BBB permeability↓ P‐gp↑ Transport vesicles↑	[137]
Spontaneous hypertensive rats	Transient middle cerebral artery occlusion	VNS	BBB permeability↓ MMPs‐2/9↓ TJ proteins↑	[138]
Baboons	Genetic generalized epilepsy	VNS	CBF in subcortex↑	[139]
Wistar rats	Irritable bowel syndrome	VNS	BBB permeability↓	[140]
Lewis rats	Experimental autoimmune encephalomyelitis	VNS	BBB permeability↓	[141]
SD rats	Ischemia–reperfusion	VNS	BBB permeability↓	[142]
SD rats	Traumatic brain injury	TNS	Low‐frequency CBF oscillations	[143]
SD rats	Healthy and subarachnoid hemorrhage	TNS	CBF↑	[144]
SD rats	Healthy	TNS	BBB permeability↑	[145]
C57BL/6 mice	Traumatic brain injury	TNS	BBB permeability↓	[146]

Abbreviations: AQP‐4, aquaporin‐4; BBB, blood–brain barrier; CBF, cerebral blood flow; MMPs, matrix metalloproteinases; P‐gp, P‐glycoprotein; TJ, tight junction; TNS, trigeminal nerve stimulation; VNS, vagus nerve stimulation.

### 6.1 Vagus Nerve Stimulation (VNS)

VNS is a neuromodulation technique that delivers electrical signals to the vagus nerve, initially proposed over a century ago by neuroscientist Corning [21]. The vagus nerve's afferent fibers transmit sensory signals from the body to the brain, primarily terminating in the nucleus tractus solitarius (NTS) of the brainstem. Due to its anatomical proximity, the NTS projects directly to LC via monosynaptic projections, allowing VNS to stimulate NE release by enhancing LC activity [147]. By observing the LC through functional magnetic resonance imaging (fMRI) and detecting the NE level in cerebrospinal fluid (CSF), existing studies have proved that VNS can activate the LC–NE system [148, 149].

Studies have demonstrated that VNS can regulate CBF and promote NVC in disease states, although its specific role remains controversial [129, 150]. For instance, in epilepsy patients, both high‐frequency and low‐frequency VNS can reduce rCBF in cortical regions and increase it in subcortical regions, particularly in the thalamus and cerebellum [125, 126, 128, 139]. In depression patients, VNS can increase the rCBF in the lateral orbitofrontal cortex and reduce it in regions such as the right dorsal anterior cingulate [151]. These changes in CBF are associated with the alleviation of the disease [125, 151]. For the ischemic stroke model, the changes in CBF caused by VNS are not significant [152]. Additionally, VNS reduces BBB permeability in conditions such as ischemic stroke, multiple sclerosis, and epilepsy [137, 138, 141].

Currently, the US Food and Drug Administration (FDA) has approved VNS for the treatment of depression, epilepsy, and ischemic stroke [21]. Furthermore, research suggests that VNS holds potential for treating various neurological disorders, including AD, PD, traumatic brain injury, and sleep disorders [136, 153, 154, 155]. Continued exploration of VNS could lead to significant advancements in the management of these neurological disorders.

### 6.2 Trigeminal Nerve Stimulation (TNS)

TNS is an innovative neuromodulation technique that delivers electrical signals to the trigeminal nerve, influencing several key brain regions, including the rostral ventrolateral nucleus (RVLM), catecholaminergic area (C1), LC, and raphe nuclei (RN) [156, 157, 158].

Research has shown that TNS can increase CBF in both healthy and pathological conditions such as epilepsy and migraine [131, 159]. Additionally, TNS exerts a bidirectional effect on BBB permeability: in healthy animal models, electroacupuncture TNS on the infraorbital nerve increases BBB permeability [145], whereas in a rat model of traumatic brain injury, TNS application to the anterior ethmoidal nerve reduces BBB permeability [146, 160].

Clinically, TNS is primarily utilized for treating chronic brain disorders such as attention deficit hyperactivity disorder in children, an indication approved by the FDA [161]. Moreover, TNS is employed to address sensorimotor and cognitive dysfunctions resulting from mild traumatic brain injury, multiple sclerosis, and cerebral palsy [162, 163, 164]. Notably, TNS not only demonstrates promise in managing chronic brain diseases but also exhibits significant effects in hyperacute brain protection, particularly in conditions like traumatic brain injury, hemorrhagic shock, subarachnoid hemorrhage, and ischemic stroke [159]. This highlights the broad therapeutic potential of TNS across various acute and chronic neurological conditions.

### 6.3 Norepinephrine Reuptake Inhibitors (NRIs)

NRIs are a class of drugs designed to block the action of the norepinephrine transporter (NET), thereby preventing the reabsorption of NE into presynaptic neurons. This mechanism ensures that more NE remains available in the synaptic cleft to exert its effects, including influencing NVU function. Common NRIs include reboxetine, atomoxetine, and atomoxetine [165].

While direct experimental evidence is limited, clinical observations suggest that NRIs can modulate CBF. For instance, in healthy individuals, the NRI atomoxetine has been shown to decrease rCBF in the midbrain, substantia nigra, and thalamic regions, while increasing rCBF in most areas of the cerebellar cortex [132]. Additionally, in patients with attention deficit hyperactivity disorder, NRIs significantly improve CBF in the prefrontal cortex [133, 134, 135]. Despite these insights, relatively few studies have examined the effects of NRIs on BBB permeability. However, in vitro BBB models indicate that NRIs like reboxetine exhibit inhibitory activity against P‐glycoprotein, suggesting they may alter BBB permeability [166]. Further research is required to elucidate the specific mechanisms involved.

Therapeutically, NRIs have a long history of use in the central nervous system for various clinical conditions, including schizophrenia, childhood attention deficit hyperactivity disorder, and depression [167, 168].

## 7 Limitations and Prospects

There is no doubt that current research has limitations. The effects of the LC–NE system on the NVU vary under different conditions. For instance, under inflammatory and non‐inflammatory conditions, the LC–NE system has different regulatory effects on astrocytic inflammation factor release [14]. Sensory stimulation alters the effect of the LC‐NE system on CBF across different brain regions [12]. Moreover, while the LC–NE system increases BBB permeability under physiological conditions, it decreases permeability under pathological conditions [145, 146]. These phenomena are not fully understood with existing research, and further exploration of the underlying mechanisms is crucial for comprehending the LC–NE system's role in maintaining brain environmental homeostasis. The bulk release of NE and the wide distribution of ARs mean that the LC–NE system's overall effect on the NVU is a competitive outcome of NE's diverse actions on various cellular receptors. Identifying which cell classes and receptors the LC–NE system preferentially targets, understanding how its action on one cell type affects others in the NVU, and elucidating how these interactions contribute to overall NVU function are essential for a detailed understanding of LC–NE system regulation.

Advancements in technology provide tools for deeper research. Using neurotransmitter probes allows for real‐time and accurate detection of the dynamic changes in neurotransmitter concentrations [169], while two‐photon imaging technology can precisely observe the morphology and functions of NVU cells [3]. At the same time, if the optogenetic techniques can be combined to activate neurons, it is possible to explore the changes in the morphology and functions of NVU cells during NVC under different release patterns of NE in different brain regions [170]. Using acute brain sectioning technology to prepare living brain slices, using drugs to simulate changes in the concentrations of various neurotransmitters, and combining two‐photon imaging to observe different levels of NVU is helpful for exploring the specific effects of various neurotransmitters on NVU when they coexist [86, 87]. In addition, constructing 3D microfluidic organ chips of brain arteries, arterioles, capillaries, and veins in vitro to simulate different levels of NVU is also a good exploration method [171]. For further exploration of the regulatory mechanisms of NE on different levels of NVU, reverse genetics (such as specific knockout/knockdown of ARs) is an ideal strategy [116]; while the use of high‐precision imaging equipment and labeling techniques (such as the genetic‐targeted neuron‐astrocyte proximity assay to measure the spatial interaction between astrocytes and neurons at the synaptic scale) can deeply analyze the internal cellular interaction mechanism of NVU in the presence of NE [172].

In the clinical application field, more evidence is still needed to enhance the clinical relevance of the connection between the LC–NE system and the NVU. The combined application of techniques for detecting the function of the LC–NE system and the NVU is a necessary research method. For the LC–NE system, functional fMRI can visualize the activation of the LC–NE system by showing the blood flow in the LC region [173]. The activity of the LC–NE system can also be measured using liquid markers such as norepinephrine and its metabolite 3‐methoxy‐4‐hydroxyphenylethanol (MHPG) in blood or CSF, or indirect indicators such as pupil dilation and cortical oscillations [174, 175]. For the NVU, methods for monitoring CBF include single photon emission computed tomography and near‐infrared spectroscopy sensors [129, 131]. Methods for assessing blood–brain barrier leakage include dynamic contrast‐enhanced MRI (DCE‐MRI) using gadolinium contrast agents, glucose chemical exchange saturation transfer imaging (glucoCEST), and arterial spin labeling (ASL) by measuring water exchange [176, 177, 178]. More strictly controlled animal studies (sham control, randomization, and appropriately blinded design) have greater validity than human clinical trials to further verify whether physical stimulation therapies and noradrenergic drugs improve NVU function through the LC–NE system. The synergistic effects of these treatments on other neural pathways also need to be considered. In addition, the application parameters of physical stimulation therapies are different. How to select appropriate parameters to maximize the repair effect of the LC–NE system on NVU requires further investigation. The widespread effects of noradrenergic drugs on systemic ARs and adverse effects limit their clinical use. Developing noradrenergic drugs targeting specific receptors in particular cells could enhance their therapeutic potential for brain diseases.

## 8 Conclusions

Numerous experimental studies have shown that the LC–NE system significantly influences the structure and function of the NVU. The activation degree and firing patterns of LC–NE neurons, as well as the release amount and action sites of NE, profoundly impact the neurons, glial cells, and cerebrovascular components of the NVU. This comprehensive regulation includes modulating CBF and BBB permeability. Clinically, treatments that activate the LC–NE system have improved NVU dysfunction in various neurological diseases, offering a reference for future therapies.

## Author Contributions

**Zixin Pan:** writing – original draft, software, resources, methodology, investigation. **Zhaoxing Jia:** resources, investigation. **Tianxiang Jiang:** resources, investigation. **Qian Cai:** resources, investigation. **Zhong Di:** resources, investigation. **Lin Gan:** writing – review and editing, supervision, methodology, conceptualization. **Congcong Ma:** writing – review and editing, supervision, methodology, conceptualization. **Xianming Lin:** writing – review and editing, supervision, methodology, conceptualization.

## Ethics Statement

The authors have nothing to report.

## Consent

The authors have nothing to report.

## Conflicts of Interest

The authors declare no conflicts of interest.

## Data Availability

The authors have nothing to report.
